# A Reference Pan-Genome Approach to Comparative Bacterial Genomics: Identification of Novel Epidemiological Markers in Pathogenic *Campylobacter*

**DOI:** 10.1371/journal.pone.0092798

**Published:** 2014-03-27

**Authors:** Guillaume Méric, Koji Yahara, Leonardos Mageiros, Ben Pascoe, Martin C. J. Maiden, Keith A. Jolley, Samuel K. Sheppard

**Affiliations:** 1 Institute of Life Science, College of Medicine, Swansea University, Swansea, United Kingdom; 2 Institute of Medical Science, University of Tokyo, Minato-ku, Tokyo, Japan; 3 Department of Zoology, University of Oxford, Oxford, United Kingdom; Charité-University Medicine Berlin, Germany

## Abstract

The increasing availability of hundreds of whole bacterial genomes provides opportunities for enhanced understanding of the genes and alleles responsible for clinically important phenotypes and how they evolved. However, it is a significant challenge to develop easy-to-use and scalable methods for characterizing these large and complex data and relating it to disease epidemiology. Existing approaches typically focus on either homologous sequence variation in genes that are shared by all isolates, or non-homologous sequence variation - focusing on genes that are differentially present in the population. Here we present a comparative genomics approach that simultaneously approximates core and accessory genome variation in pathogen populations and apply it to pathogenic species in the genus *Campylobacter*. A total of 7 published *Campylobacter jejuni* and *Campylobacter coli* genomes were selected to represent diversity across these species, and a list of all loci that were present at least once was compiled. After filtering duplicates a 7-isolate reference pan-genome, of 3,933 loci, was defined. A core genome of 1,035 genes was ubiquitous in the sample accounting for 59% of the genes in each isolate (average genome size of 1.68 Mb). The accessory genome contained 2,792 genes. A *Campylobacter* population sample of 192 genomes was screened for the presence of reference pan-genome loci with gene presence defined as a BLAST match of ≥70% identity over ≥50% of the locus length - aligned using MUSCLE on a gene-by-gene basis. A total of 21 genes were present only in *C. coli* and 27 only in *C. jejuni*, providing information about functional differences associated with species and novel epidemiological markers for population genomic analyses. Homologs of these genes were found in several of the genomes used to define the pan-genome and, therefore, would not have been identified using a single reference strain approach.

## Introduction

Periodic advances in DNA sequencing technology, such as wide-spread adoption of automated DNA sequencing in the 1990s, have revolutionized understanding of microbial processes, from single-cell physiology to population biology [1], [2]. The last decade saw the increased use of high-throughput or ‘next-generation’ sequencing methods that parallelize the DNA sequencing process beyond what was possible with standard dye-terminator methods. These technologies have underpinned important research in pathogen epidemiology and evolution [3], [4], [5], [6], [7], [8], but there are still major technical challenges for effectively archiving and analyzing hundreds or thousands of bacterial genomes [9].

A popular approach to describe the genetic variation among multiple bacterial genomes has been to map stretches of DNA sequences from multiple isolates to a reference bacterial genome to identify variable sites that display single nucleotide polymorphisms (SNPs). This is an effective way of condensing large genomes into panels of informative sites. This has provided detailed information on the genetic structure and transmission of pathogen species with relatively low sequence diversity, such as *Mycobacterium tuberculosis* [10] or *Yersinia pestis* [11], and for single lineages of more diverse species, for example *E. coli* O157:H7 [12]. However, this approach has potential limitations, particularly when applied to highly diverse species such as *Campylobacter jejuni*. First, because it requires careful separation of biologically informative SNPs from relatively common sequencing errors, and second because this approach typically treats dispersed and locally clustered SNPs equally even though the later are likely to be the consequence of horizontal genetic exchange.

An alternative to using a reference genome SNP-based approach is to use genes as the units of comparison. In this reference gene based approach [13], genetic variation within the sample is catalogued one gene at a time by comparison with reference gene sequences, and each new variant is assigned a unique arbitrary allele number in order of description. This whole-genome multilocus sequence typing (MLST) approach enables locus information to be defined in simultaneously in hundreds of genomes and has been implemented for genera, including *Campylobacter*, *Staphylococcus*, and *Neisseria*, using the web-based BIGSdb platform (http://zoo-talisker.zoo.ox.ac.uk/dbases/ [14], [15]).

Both the SNP-based and gene-by-gene approaches rely on reference-based mapping and cannot be used to detect variation in genes that are not present in the reference isolate sequence or locus list. This is not important in analyses based on comparison of a core genome shared among all isolates, but may be less suitable for the discovery of novel genes and functions, and for the examination of the accessory genome composed of genes that vary in presence across isolates of the same population. Here we address this challenge by combining multiple reference genomes to create a single list of genes present in 7 reference genomes from which gene presence and variation can be examined in other bacterial genomes. This list of genes will be hereafter termed the ‘reference pan-genome’ - not to be confused with the true pan-genome as it is based on just 7 isolates. This technique is then applied to characterize the genetic variation in *Campylobacter jejuni* and *Campylobacter coli*.

*C. jejuni* and *C. coli* are common constituents of the commensal gut microbiota of various bird and mammal species. Human infection, typically associated with the consumption of contaminated meat or poultry [16], [17], results in symptoms of severe diarrhea and fever. Campylobacteriosis is currently the most common form of bacterial gastroenteritis in industrialized countries, accounting for an estimated 1 million cases in the UK each year [18], with an annual economic burden of £500 million [19]. In spite of its public health importance, aspects of the ecology and evolution of *Campylobacter* remain poorly understood, even though they could have a profound effect on transmission and human infection. For example, it is not fully explained how *C. coli* and *C. jejuni*, that have similar host niches and frequently exchange genetic material [20], [21], [22], differ in terms of their disease epidemiology. Furthermore, within *C. jejuni* there are lineages that are largely limited to one host and others that are frequently isolated from multiple hosts and are common in human disease [7], [23], [24]. This ecological variation will have an impact on transmission ecology in *C. coli* and *C. jejuni* and here we aim to define the genomic differences between species and lineages and identify informative epidemiological markers using a reference pan-genome approach.

## Materials and Methods

### Characterizing the reference pan genome

**Figure 1:**
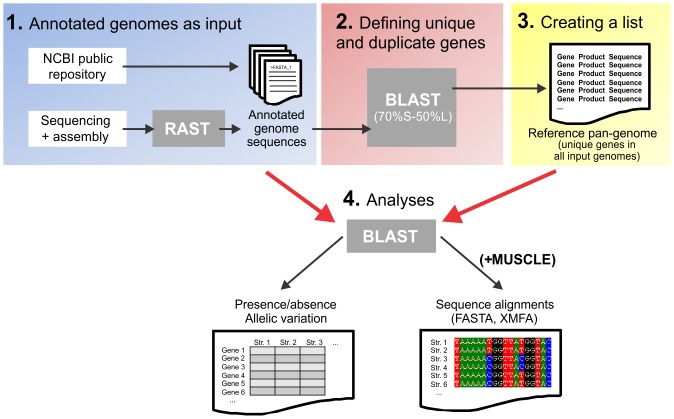
The reference pan-genome approach. Conceptual pipeline showing the approach to generate a list of unique genes from more than one reference strain. Step 1: Compiling a gene list from reference genomes reflecting strain diversity from public repositories such as NCBI, or after automatic annotation on assembled contiguous segments (for example using RAST). Step 2: Comparative list analysis to remove duplicate genes that show ≥70% sequence identity over ≥50% of the sequence of another gene in the list. Step 3: Creating a final reference pan-genome list.

The reference pan-genome approach combines the genomes of several reference strains into a single list of genes for those isolates. This gene list was then used for genome comparisons with the larger sample collection (**Figure 1**). Different numbers of reference strains can be used depending on the genome size and diversity of the accessory genome, but it is important to note that the pan-genome size will influence computation time for downstream applications. For species where finished annotated genomes are not available the reference pan-genome list can be assembled from whole genome contiguous sequence files from several isolates with automatic annotation, for example using RAST [25]. The list of genes from the various reference genomes was then screened to remove genes that appeared more than once, to create the reference pan-genome. Homologous genes were defined using BLAST as those that had >70% sequence similarity over >50% of the sequence length to another gene in the list. In *Campylobacter*, the average core genome nucleotide sequence divergence between *C. jejuni* and *C. coli* is around 12.5% [6], corresponding to approximately 87.5% nucleotide sequence identity which is considerably higher the BLAST match criteria. Duplicate genes were then removed. The BLAST threshold can be altered depending on the bacterial species used and on the desired stringency.

To most effectively capture genetic variation within *C. coli* and *C. jejuni*, and therefore construct a representative reference pan-genome, lineages were selected to represent the known genetic diversity based on published genealogies (**Table 1** [6], [7]). In *Campylobacter*, annotated published reference genomes were available for both *C. coli* and *C. jejuni* that reflected diversity. The resulting *Campylobacter* reference pan-genome was based upon 7 published genomes: *C. jejuni subsp. jejuni* strains NCTC11168 [26], 81–176 [27], 81116 [28] and M1 [29]; *C. jejuni subsp. doylei* strain 269.97 (Genbank: NC_009707.1); *C. coli* strains 76339 (Genbank: NC_022132.1) and CVM N2970 [30] (**Table 1**). These genomes included both *C. coli* and *C. jejuni* species, two *C. jejuni* subspecies (*jejuni* and *doylei*) and 6 clonal complexes defined as sharing 4 or more identical alleles at 7 MLST housekeeping gene loci.

**Table 1:** Publicly-available genomes used to produce a *Campylobacter* reference pan-genome.

Strain name	Lineage	Annotated genes	Genome size (Mbp)	NCBI Accession
*C. jejuni subsp. jejuni* NCTC11168	ST-21 complex	1,670	1.64	NC_002163.1
*C. jejuni subsp. jejuni* 81-176	ST-42 complex	1,812	1.7	NC_008787.1
*C. jejuni subsp. jejuni* 81116	ST-283 complex	1,681	1.63	NC_009839.1
*C. jejuni subsp. jejuni* M1	ST-45 complex	1,675	1.62	NC_017280.1
*C. coli* 76339	Clade 3	1,556	1.58	NC_022132.1
*C. coli* CVM N29710	Clade 1	1,747	1.73	NC_022347.1
*C. jejuni subsp. doylei* 269.97	ST-1845	2,037	1.85	NC_009707.1
Total size	-	12,178	11.75	-
*Campylobacter* reference pan-genome size	-	3,933	3.72	-

### Reference pan-genome analyses

Genetic variation at pan-genome loci was investigated in 192 *C. jejuni* and *C. coli* genomes from previously published studies (**Table S1** [6], [7]). These isolate genomes were compared to the pan-genome locus list using BLAST. Variation within the population genomic sample was catalogued one gene at a time with gene presence defined as a match with >70% sequence identity to >50% of a locus. The result was a matrix recording the presence or absence of each gene by comparison with reference pan-genome gene sequences and each new gene sequence variant was assigned a unique arbitrary allele number in order of description. These analyses were implemented in the BIGSdb database platform [13], [14].

### Rarefaction and accumulation curves

The rarefaction and accumulation curves for core and pan-genome size estimations were created using a R software script (**File S1**), and were inferred from a matrix of presence/absence of loci from the reference pan-genome list in all 192 genomes. The input was a matrix of gene presence and absence of the 7-isolate reference pan-genome. These genes were identified in the 192 sample genomes by BLAST comparsion (as described above). The number of core genes shared by all isolates, and the cumulative number of different genes was calculated as the number of genomes sampled increased. Randomized calculations were carried out with 100 repeats, randomizing the order of the genomes each time to obtain mean core and pan genome size estimates and standard errors.

### Phylogenetic analyses

**Figure 2:**
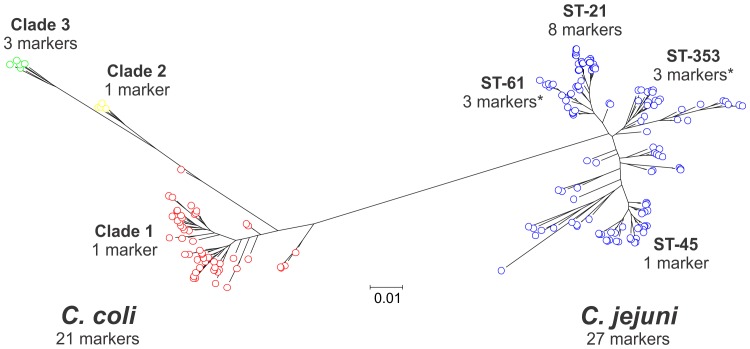
Phylogenetic tree of 192 *Campylobacter* genomes and novel epidemiological markers. Maximum-likelihood tree of 130 *C. jejuni* and 62 *C. coli* genomes. Isolates belonging to *C. jejuni* are shown in blue, and those belonging to *C. coli* clade 1 are indicated in red, clade 2 in yellow, and clade 3 in green. The scale bar indicates the estimated number of substitutions per site. Example genomes from *C. coli* clades 1-3 and C. jejuni ST-21, ST-45, ST-353 and ST-61 clonal complexes were used to define the 7 isolate reference pan-genome gene list. The number of epidemiological markers from this list is indicated for each lineage. The asterisk indicates that markers were not found to be absolutely specific to that lineage, but were also present at low frequency in other lineages. Details about the markers are shown in Table 2 and Table 3.

Gene homologs were aligned on a gene-by-gene basis using MUSCLE [31] and then concatenated into contiguous sequence for each isolate genome including gaps for missing nucleotides (or entire genes) (**File S2**). A phylogeny of core genome alignments was reconstructed using FastTree v2.1.7 [32] with an approximation of the maximum-likelihood algorithm (**Figure 2**). The tree was created using 61,844 variable sites for a total of 378,003 shared sites from 1,035 loci.

## Results and Discussion

### Core and accessory genome variation

**Figure 3:**
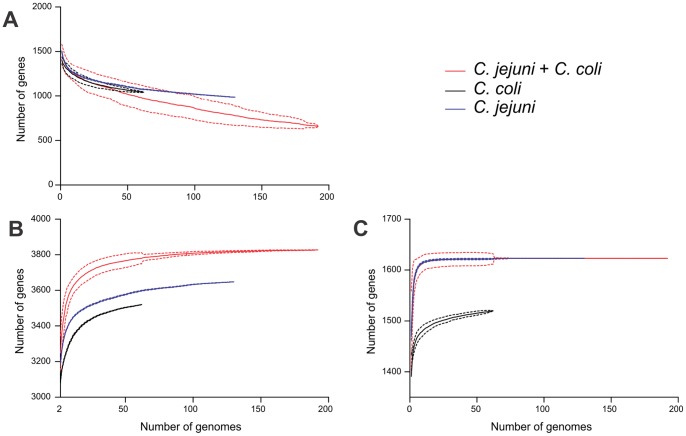
Rarefaction and accumulation curve estimates of *C. jejuni* and *C. coli* core and pan-genomes. The number of shared genes (A), and the total number of genes (B and C), were determined as genome sampling increased. Comparisons were made based on matrices of gene presence/absence, derived from the reference pan-genome list, for *C. coli* (62 genomes), *C. jejuni* (130 genomes) and the two species combined (192 genomes). Randomized genome sampling was carried out 100 times to obtain the average number of genes for each sample comparison number (plain lines) and standard deviations (dotted lines). Pan-genome size estimates were calculated using the reference pan-genome (B) or the NCTC11168 annotation (C).

The 7 reference genomes used to assemble the reference pan-genome list contained 12,178 genes with a total length of more than 11 Mbp. The resulting pan-genome list, after removal of duplicate genes present in more than one reference genome, consisted of 3,933 genes, with a total coding sequence length of 3.72 Mbp (**Table 2**). There were 8,245 duplicate genes. Core and pan-genome sizes were estimated using rarefaction and accumulation curves (**Figure 3**). As expected, the number of genes shared by all isolates (**Figure 3A**) decreased as the number of sample genomes in the dataset increased, with 660 genes present in all 192 genomes. The estimated core genome of *C. coli* was 1,042 genes in 62 genomes. The estimated core genome of *C. jejuni* was 947 genes in 130 genomes (**Figure 3A**). Our estimates are consistent with previous studies where core genome size of *C. jejuni* was estimated to range from 847 genes [33] and 1,001 genes [34] to a maximum of 1,295 genes [29]. However, it is interesting to note that the core genome size does not reach a clear plateau, even when about 200 genomes are sampled, which indicates that if more diverse samples were added to this analysis, even fewer genes would be shared, something that has also been shown for *Escherichia coli* [35].

**Table 2:** Functional categories of genes present in the reference pan-genome and in the reference genome of *C. jejuni* NCTC11168.

Functional category	Reference pan-genome	*C. jejuni* NCTC11168
Protein Metabolism	328 (12.2%)	213 (14.9%)
Cell Wall and Capsule	318 (11.8%)	125 (8.7%)
Cofactors, Vitamins, Prosthetic Groups, Pigments	285 (10.6%)	134 (9.4%)
Amino Acids and Derivatives	283 (10.5%)	181 (12.6%)
Virulence, Disease and Defense	167 (6.2%)	67 (4.7%)
Respiration	155 (5.8%)	72 (5.0%)
Motility and Chemotaxis	155 (5.8%)	86 (6.0%)
DNA Metabolism	148 (5.5%)	66 (4.6%)
RNA Metabolism	132 (4.9%)	65 (4.5%)
Carbohydrates	111 (4.1%)	62 (4.3%)
Membrane Transport	106 (3.9%)	51 (3.6%)
Iron acquisition and metabolism	96 (3.6%)	43 (3.0%)
Fatty Acids, Lipids, and Isoprenoids	92 (3.4%)	64 (4.5%)
Stress Response	76 (2.8%)	42 (2.9%)
Nucleosides and Nucleotides	63 (2.3%)	52 (3.6%)
Cell Division and Cell Cycle	38 (1.4%)	21 (1.5%)
Regulation and Cell signaling	33 (1.2%)	16 (1.1%)
Phosphorus Metabolism	30 (1.1%)	20 (1.4%)
Nitrogen Metabolism	21 (0.8%)	13 (0.9%)
Potassium metabolism	20 (0.7%)	16 (1.1%)
Regulons	12 (0.4%)	5 (0.3%)
Secondary Metabolism	7 (0.3%)	4 (0.3%)
Sulfur Metabolism	5 (0.2%)	5 (0.3%)
Miscellaneous	4 (0.1%)	4 (0.3%)
Metabolism of Aromatic Compounds	3 (0.1%)	3 (0.2%)
Dormancy and Sporulation	1 (0.0%)	1 (0.1%)
**Total number of genes assigned to a known function**	**2689**	**1431**

The pan-genome size was characterized for *C. jejuni* and *C. coli* by quantifying the number of reference pan genome genes as the number of sample genomes increased. The total pan-genome for 130 *C. jejuni* genomes contained 3,648 genes (**Figure 3B**). Ninety-two percent of this total was identified from comparison of only 7 genomes (3,388±31 genes), and 99% of the pan-genome size estimate was reached after comparing 75 genomes (**Figure 3B**). The pan-genome was smaller in *C. coli* with an estimated 3,520 genes identified in 62 sample genomes. A very similar proportion of the pan-genome genes were detected by comparison of 7 genomes (93%, 3267±50 genes), and comparison of 40 genomes captured 99% of the total reference pan-genome (**Figure 3B**). When a single reference genome comparison was used, rather than the 7 isolate reference, the pan genome was greatly underestimated (**Figure 3C**). Almost all genes present in 11168 were found to be present in just 5 sample genomes of *C. jejuni* and *C. coli* (**Figure 1C**).

Comparative genomics approaches based on the alignment to a single genome will ignore genetic variation that is not present in this reference genome. For example, the *C. jejuni* strain NCTC11168 - which has a well annotated genome [26] - is commonly used in comparative genome studies [6], [7]. This strain belongs to the ST-21 clonal complex, and while 93% (1,521/1,623) of its genes are present in all ST-21 clonal complex isolates, this number drops to 88% (1,424/1,623) for ST-45 clonal complex isolates and 69% (1,121/1,623) for *C. coli* Clade 1 isolates. Core genome analyses may not be affected by this issue, as only the shared genes between all strains are examined. However, 263 accessory genes, identified using the reference pan-genome approach in this study, are absent in *C. jejuni* 11168 but present in the ST-45 complex. Amongst this accessory genome there could be genes associated with important adaptive traits such as virulence or colonization factors linked to metabolism or host-association [7], [36].

### Variation in functional gene categories

By investigating variation in functional categories of genes in the reference pan-genome, some inference can be made about putative phenotype differences between species and lineages. To maximize the available genome annotation information beyond that which is available for the *C. jejuni* NCTC11168 isolate [26], all the genes comprising the pan-genome were concatenated and submitted to the RAST automatic annotation server, to attribute putative function (**Table 2**). From a total of 1,623 genes, 1,431 (88%) were assigned to functional categories. Among these, the categories with most genes were associated with protein metabolism, the cell wall and capsule, cofactors and vitamins and amino-acids and derivatives (**Table 2**). The proportions of the various functional categories attributed by RAST to the reference pan-genome list were different from those of *C. jejuni* NCTC11168 (χ^2^ = 40.09, d.f. = 25; p = 0.0286). Higher proportions for genes involved in cell wall and capsule and virulence factors were found in the pan-genome compared to *C. jejuni* NCTC11168, indicating that these genes functions are better represented in the pan-genome gene list.

### Lineage specific genes in *C. jejuni* and *C. coli*

Comparison of patterns of gene presence/absence in the pan-genome genes of 192 *C. jejuni* and *C. coli* genomes was performed to identify genes that segregated by species or lineage. Segregation was either: complete, with genes present in one group and absent in the other; or frequency dependent, where genes were significantly more frequent in one lineage. Consistent with the aim of discovering informative epidemiological markers, we focused on accessory genes that were the most specific to each of the 7 examined lineages of *C. coli* and *C. jejuni*. Of the 3,933 genes of the pan-genome, there were 20 genes specific to each of the 7 lineages (**Figure 2**).

Forty-eight genes were found to be differentially present in the species *C. jejuni* and *C. coli* (**Table 3**). It is interesting to note that the genes present in *C. coli* (62/62, 100%) but not *C. jejuni* (0/130, 0%) are all present in the *C. coli* 76339 reference genome used to compile the pan-genome, and none were present in the *C. jejuni* reference strain NCTC 11168. This highlights the fact that if a typical single strain reference approach, based on a NCTC 11168, had been used to identify genetic markers specific to *C. coli*, all of these genes would have been missed. Twenty-seven genes were found to be present in all *C. jejuni* (130/130, 100%) and absent in all *C. coli* (0/62, 0%).

**Table 3:** Prevalence of *C. coli* and *C. jejuni* associated genes from a comparison of 192 genomes.

Gene identifier	Description	Detailed functional categories	Gene prevalence									Species association
			*C. coli*			*C. jejuni* clonal complex				All *C. coli* (n = 62)	All *C. jejuni* (n = 130)	
			Clade 1 (n = 47)	Clade 2 (n = 4)	Clade 3 (n = 5)	ST-21 (n = 41)	ST-45 (n = 28)	ST-353 (n = 7)	ST-61 (n = 6)			
Cc76339__00005c	Methyl-accepting chemotaxis protein, putative	-	47	4	5	0	0	0	0	62	0	*C. coli*
Cc76339__01340	Cytolethal distending toxin subunit C	Cytolethal distending toxins	47	4	5	0	0	0	0	62	0	*C. coli*
Cc76339__01460c	2-methylcitrate dehydratase (EC 4.2.1.79)	Methylcitrate cycle	47	4	5	0	0	0	0	62	0	*C. coli*
Cc76339__01470c	2-methylcitrate synthase (EC 2.3.3.5)	Methylcitrate cycle	47	4	5	0	0	0	0	62	0	*C. coli*
Cc76339__01480c	Methylisocitrate lyase (EC 4.1.3.30)	Methylcitrate cycle	47	4	5	0	0	0	0	62	0	*C. coli*
Cc76339__01490c	Propionate—CoA ligase (EC 6.2.1.17) / Acetyl-coenzyme A synthetase (EC 6.2.1.1)	Methylcitrate cycle; Pyruvate metabolism	47	4	5	0	0	0	0	62	0	*C. coli*
Cc76339__01750	Highly acidic protein	-	47	4	5	0	0	0	0	62	0	*C. coli*
Cc76339__02240	Integral membrane protein TerC	-	47	4	5	0	0	0	0	62	0	*C. coli*
Cc76339__03250	hypothetical protein	-	47	4	5	0	0	0	0	62	0	*C. coli*
Cc76339__04670	probable periplasmic protein Cj0093, putative	-	47	4	5	0	0	0	0	62	0	*C. coli*
Cc76339__09670	Hypothetical protein Cj1162c	-	47	4	5	0	0	0	0	62	0	*C. coli*
Cc76339__10710	Small hydrophobic protein	-	47	4	5	0	0	0	0	62	0	*C. coli*
Cc76339__10950	FIG00469427: hypothetical protein	-	47	4	5	0	0	0	0	62	0	*C. coli*
Cc76339__11130	Putative periplasmic protein	-	47	4	5	0	0	0	0	62	0	*C. coli*
Cc76339__11470	Uncharacterized protein Cj0990c	-	47	4	5	0	0	0	0	62	0	*C. coli*
Cc76339__11500c	Surface-exposed lipoprotein JlpA	Adhesion	47	4	5	0	0	0	0	62	0	*C. coli*
Cc76339__12660c	Zinc ABC transporter, periplasmic-binding protein ZnuA	-	47	4	5	0	0	0	0	62	0	*C. coli*
Cc76339__12670	Peroxide stress regulator / Ferric uptake regulation protein	Oxidative stress; Iron Metabolism	47	4	5	0	0	0	0	62	0	*C. coli*
Cc76339__12940	CoA-binding domain protein	-	47	4	5	0	0	0	0	62	0	*C. coli*
Cc76339__15800	Methionine synthase II (cobalamin-independent)	-	47	4	5	0	0	0	0	62	0	*C. coli*
Cc76339__15900c	FIG00469900: hypothetical protein	-	47	4	5	0	0	0	0	62	0	*C. coli*
11168_Cj0011c	Periplasmic dsDNA and ssDNA-binding protein contributing to transformation	-	0	0	0	41	28	7	6	0	130	*C. jejuni*
11168_Cj0090	Putative lipoprotein	-	0	0	0	41	28	7	6	0	130	*C. jejuni*
11168_Cj0135	Hypothetical protein Cj0135	-	0	0	0	41	28	7	6	0	130	*C. jejuni*
11168_Cj0186c	Integral membrane protein TerC	-	0	0	0	41	28	7	6	0	130	*C. jejuni*
11168_Cj0327	Putative translation initiation inhibitor, yjgF family	-	0	0	0	41	28	7	6	0	130	*C. jejuni*
11168_Cj0339	Putative transmembrane transport protein	-	0	0	0	41	28	7	6	0	130	*C. jejuni*
11168_Cj0340	Inosine-uridine preferring nucleoside hydrolase (EC 3.2.2.1)	Purine conversions; Queuosine-Archaeosine Biosynthesis	0	0	0	41	28	7	6	0	130	*C. jejuni*
11168_Cj0414	FIG00471287: hypothetical protein	-	0	0	0	41	28	7	6	0	130	*C. jejuni*
11168_Cj0454c	membrane protein	-	0	0	0	41	28	7	6	0	130	*C. jejuni*
11168_Cj0494	FIG00469900: hypothetical protein	-	0	0	0	41	28	7	6	0	130	*C. jejuni*
11168_Cj0873c	Cytochrome c family protein	-	0	0	0	41	28	7	6	0	130	*C. jejuni*
11168_Cj0900c	Small hydrophobic protein	-	0	0	0	41	28	7	6	0	130	*C. jejuni*
11168_Cj1021c	Putative periplasmic protein	-	0	0	0	41	28	7	6	0	130	*C. jejuni*
11168_Cj1036c	FIG00469427: hypothetical protein	-	0	0	0	41	28	7	6	0	130	*C. jejuni*
11168_Cj1060c		-	0	0	0	41	28	7	6	0	130	*C. jejuni*
11168_Cj1162c	Hypothetical protein Cj1162c	-	0	0	0	41	28	7	6	0	130	*C. jejuni*
11168_Cj1666c	CopG protein	Copper homeostasis	0	0	0	41	28	7	6	0	130	*C. jejuni*
11168_Cj1714		-	0	0	0	41	28	7	6	0	130	*C. jejuni*
11168_ctsT	Transformation system protein	-	0	0	0	41	28	7	6	0	130	*C. jejuni*
11168_kdpD	Osmosensitive K+ channel histidine kinase KdpD (EC 2.7.3.-)	Potassium homeostasis	0	0	0	41	28	7	6	0	130	*C. jejuni*
11168_tonB2	Ferric siderophore transport system, periplasmic binding protein TonB	Iron Metabolism	0	0	0	41	28	7	6	0	130	*C. jejuni*
Cj_81-176_1820	Putative transmembrane transport protein	-	0	0	0	41	28	7	6	0	130	*C. jejuni*
Cj_81-176_6530	FIG00469465: hypothetical protein	-	0	0	0	41	28	7	6	0	130	*C. jejuni*
Cj_81-176_8530		-	0	0	0	41	28	7	6	0	130	*C. jejuni*
Cj_81-176_8535		-	0	0	0	41	28	7	6	0	130	*C. jejuni*
Cj81116_1523		-	0	0	0	41	28	7	6	0	130	*C. jejuni*
Cjdoleyi_26997_0913	Small hydrophobic protein	-	0	0	0	41	28	7	6	0	130	*C. jejuni*

There were several genes that segregated according to 3-clade structure in *C. coli* [37] (**Figure 2**, **Table 4**). One gene (*CcCVMN29710_G157_03450*), annotated as encoding a reductase involved in fatty acid biosynthesis, was present in all *C. coli* clade 1 isolates (47/47) but was absent from isolates in the other *C. coli* clades (0/9) and from *C. jejuni* (0/130). Similarly, one gene (*Cc76339_10830*), encoding a hypothetical protein was present in *C. coli* clade 2 (4/4) but absent elsewhere (0/188). Three genes were present in all 5 genomes of *C. coli* clade 3 and absent in all other *C. coli* (0/57) and most *C. jejuni* (2/130) except for 2 environmental isolates. These genes putatively encoded a biotin sulfoxide reductase, a secreted serine protease and a cytochrome C-type periplasmic protein,

**Table 4:** Lineage associated genes in *C. coli* and *C. jejuni* from a comparison of 192 genomes.

Gene identifier	Description	Detailed functional categories	Gene prevalence									Species/clade association
			*C. coli*			*C. jejuni* clonal complex				All *C. coli* (n = 62)	All *C. jejuni* (n = 130)	
			Clade 1 (n = 47)	Clade 2 (n = 4)	Clade 3 (n = 5)	ST-21 (n = 41)	ST-45 (n = 28)	ST-353 (n = 7)	ST-61 (n = 6)			
*CcCVMN29710_G157_03450*	3-oxoacyl-[acyl-carrier protein] reductase	Fatty Acid Biosynthesis	47	4	0	0	0	0	0	53	0	*C. coli* Clade 1
*Cc76339__10830*	Hypothetical protein	-	0	4	0	0	0	0	0	4	0	*C. coli* Clade 2
*Cc76339__04060*	Biotin sulfoxide reductase	-	0	0	5	0	0	0	0	5	1	*C. coli* Clade 3
*Cc76339__07680c*	Putative secreted serine protease	-	0	0	5	0	0	0	0	5	1	*C. coli* Clade 3
*Cc76339__04070*	Putative cytochrome C-type haem-binding periplasmic protein	-	0	0	5	0	0	0	0	5	1	*C. coli* Clade 3
*11168_ald'*	Aldehyde dehydrogenase	L-rhamnose utilization	43	0	0	41	0	0	0	48	59	*C. jejuni* ST-21
*11168_Cj0480c*	Transcriptional regulator	Aromatic compound degradation	38	0	0	41	0	0	0	43	58	*C. jejuni* ST-21
*11168_Cj0485*	Putative oxidoreductase	-	43	0	0	41	0	0	0	48	59	*C. jejuni* ST-21
*11168_Cj0486*	Fucose permease	L-fucose utilization	43	0	0	41	0	0	0	48	59	*C. jejuni* ST-21
*11168_Cj0487*	Predicted metal-dependent hydrolase of the TIM-barrel fold	-	42	0	0	41	0	0	0	47	59	*C. jejuni* ST-21
*11168_Cj0488*	Hypothetical protein	-	43	0	0	41	0	0	0	48	59	*C. jejuni* ST-21
*11168_dapA*	Putative lyase	-	43	0	0	41	0	0	0	48	59	*C. jejuni* ST-21
*11168_uxaA'*	Altronate hydrolase	D-Galacturonate and D-Glucuronate Utilization	43	0	0	41	0	0	0	48	59	*C. jejuni* ST-21
*Cj81116_1569*	Putative periplasmic protein	-	0	0	0	0	28	0	0	0	48	*C. jejuni* ST-45
*Cjdoleyi_26997_0954*	Hypothetical protein	-	15	0	0	0	0	7	0	16	27	*C. jejuni* ST-353
*Cjdoleyi_26997_0958*	hypothetical protein	-	11	0	0	0	0	7	0	12	21	*C. jejuni* ST-353
*Cjdoleyi_26997_0959*	Death-on-curing family protein	-	5	0	0	0	0	7	0	6	21	*C. jejuni* ST-353
*CcCVMN29710_G157_08075*	Hypothetical protein	-	33	3	0	1	0	0	6	42	11	*C. jejuni* ST-61
*CcCVMN29710_G157_06925*	Membrane protein	-	40	1	0	2	0	0	6	46	20	*C. jejuni* ST-61
*CcCVMN29710_G157_06930*	Membrane protein	-	38	1	0	2	0	0	6	44	20	*C. jejuni* ST-61

Within *C. jejuni*, accessory gene specificity for particular lineages was not complete with every gene present in high frequency in one of the major lineages also being present in minor lineages. This is not surprising as the genetic distance between clonal complexes is less than between species or the *C. coli* clades leading to increased gene flow because of the homology dependence of recombination [38]. There were, however, genes that could be associated with the ST-21 and ST-45 clonal complexes which are frequently isolated from multiple hosts [39], and the ST-353 and ST-61 complexes that are more host restricted.

Eight genes were present in 41/41 ST-21 clonal complex isolates, absent in *C. jejuni* ST-45, ST-61 and ST-353 complexes (0/89), but present in *C. coli* clade 1 (up to 43/47 isolates). This is consistent with previously reported gene flow between *C. coli* and *C. jejuni* [6], [21]. These genes were also present in less frequent lineages of our dataset, notably ST-257 (in all 3 isolates) and ST-354 (in all 3 isolates) clonal complexes. One gene (*cj81116_1569*), encoding a putative periplasmic protein, was present at high frequency in the ST-45 clonal complex (28/28 isolates) and was absent from all other *C. jejuni C*. and *C. coli* isolates. The genes that are differentially present in ST-21 and ST-45 clonal complexes, provide support to the idea that while these lineages occupy the same hosts, they may have characteristics that differentiate them.

There were fewer lineage-specific genes in the chicken and cattle host associated ST-353 and ST-61 clonal complexes. Three genes were found to be present in all ST-353 clonal complex isolates (7/7, 100%) and not in the other most common *C. jejuni* clonal complexes in our dataset (**Table 3**). They were, however, present in 11(32%) of *C. coli* Clade 1 isolates and in the ST-257, ST-354, ST-508 and ST-573 clonal complexes. An interesting observation is that when these three genes were present in *C. jejuni*, it was mostly in isolates from chicken (17/21, 81%). As the ST-353 clonal complex is a chicken associated lineage [20], it can be expected that genes associated with this clonal complex may also be present in other chicken-associated lineages. Three other genes were found to be associated with the ST-61 clonal complex (**Table 4**), also without absolute specificity as the genes were commonly found in *C. coli* and some other *C. jejuni* clonal complexes.

### Functional grouping of discriminating genes

*Campylobacter* uses short-chained fatty acids as nutrients, which are typical by-products of acetate and lactate metabolism by many gastrointestinal bacteria [40]. As in other bacteria [41], [42], one of the β-oxidation by-products of fatty acids chains is metabolized via the methylcitrate cycle. Interestingly, 4 genes specifically found in *C. coli* and not *C. jejuni* were involved in the methylcitrate cycle (**Table 3**), which could highlight a preference or enhanced ability of *C. coli* strains to grow on odd-chained fatty acids compared to *C. jejuni*. With more focused development, this observation could potentially lead to the development of specific fatty-acid-rich media designed to discriminate more efficiently between *C. jejuni* and *C. coli*, or to improve isolation frequency of *C. coli* in the laboratory.

Another functional characteristic that differed between the two species was described by genes involved in copper and iron acquisition and homeostasis, which absolutely segregated between *C. jejuni* and *C. coli* in our dataset (**Table 3**). This could indicate that while these functions are important for both species, the genes involved in them are divergent, maybe indicative of convergent evolution, or compensatory systems. Additionally, we observed that the genes preferentially found in the ST-21 complex of *C. jejuni* were involved in the metabolism of various compounds (**Table 3**) such as L-rhamnose, L-fucose or aromatic compounds, as previously suggested [43]. The metabolism of L-fucose has been shown to be associated with gastrointestinal fitness in *C. jejuni* [36], but also enriched in ST-21 clonal complex isolates [43] and in introgressed Clade 1 *C. coli* [6]. This could potentially indicate that isolates from the ST-21 complex could have a higher metabolic plasticity compared to others.

## Conclusion

The reference pan-genome approach, in this case based on 7 diverse *C. jejuni* and *C. coli* isolate genomes, was useful for investigating patterns of species- and lineage-specific genetic variation. Enhanced estimates of the core and accessory genome size were possible and several genes that were differentially present in the species and lineages were identified. The genetic segregation varied among lineages and was more pronounced for the 3 *C. coli* clades than within *C. jejuni*, where absolute segregation was rarely observed because of frequent genetic exchange. However, it was possible to identify genes that may provide information about some of the putative differences between species, clades and clonal complexes. As well as informing studies based on gene function, these genes can potentially act as epidemiological markers for differentiating strains.

## Supporting Information

Table S1 **List of 192 genomes used in this study**.
(DOCX)

File S1 **Scripts to calculate core genome rarefaction and pan-genome accumulation.** The file contains R scripts and an example input file.
(ZIP)

File S2 **Core genome alignment (FASTA format) for the 192 genomes used in this study.** Core genes shared by all 192 isolates were aligned in a gene-by-gene manner (see methods) and concatenated.
(GZ) Click here for additional data file.
