# Supplementary material for: A Reference Pan-Genome Approach to Comparative Bacterial Genomics: Identification of Novel Epidemiological Markers in Pathogenic Campylobacter
Source: PLoS One. 2014 Mar 27;9(3):e92798. doi: 10.1371/journal.pone.0092798 (PMC3968026; doi:10.1371/journal.pone.0092798)
Supplement: Table S1 — List of 192 genomes used in this study. (DOCX) [file pone.0092798.s001.docx]

**Table S1.** List of 192 genomes used in this study.

| **Genome ID** | **Name** | **Assembled genome size (bp)** | **Source** | **ST** | **Clonal complex** | **Reference** |
| --- | --- | --- | --- | --- | --- | --- |
| 1 | 182 | 1,752,685 |  |  |  | [6] |
| 2 | 171 | 1,678,444 |  | 867 | ST-828 | [6] |
| 3 | 316 | 1,624,910 |  | 1992 |  | [6] |
| 4 | CAMP45 | 1,596,969 | chicken | 45 | ST-45 | [6] |
| 5 | CAMP2696 | 1,589,408 | pig | 2696 |  | [6] |
| 6 | CAMP2681 | 1,542,097 | chicken |  |  | [6] |
| 7 | CAMP1576 | 1,521,545 | chicken | 1576 |  | [6] |
| 8 | CAMP1670 | 1,547,646 | human | 1670 |  | [6] |
| 9 | CAMP1643 | 1,500,025 | human | 1643 |  | [6] |
| 10 | CAMP1572 | 1,660,889 | chicken | 1572 |  | [6] |
| 11 | CAMP2016 | 1,606,337 | duck | 2016 |  | [6] |
| 12 | CAMP2326 | 1,554,713 | human | 2326 |  | [6] |
| 13 | CAMP61 | 1,551,351 | cattle | 61 | ST-61 | [6] |
| 14 | CAMP2381 | 1,543,877 | farm and riparian | 2381 |  | [6] |
| 15 | CAMP886 | 1,594,196 | pig | 887 | ST-828 | [6] |
| 17 | CAMP2588 | 1,633,576 | chicken | 867 | ST-828 | [6] |
| 18 | CAMP3667 | 1,618,028 | chicken | 3667 | ST-1150 | [6] |
| 19 | CAMP3129 | 1,678,945 | human |  |  | [6] |
| 20 | CAMP1487 | 1,505,418 | chicken |  |  | [6] |
| 21 | CAMP1090 | 1,566,888 | chicken | 828 | ST-828 | [6] |
| 22 | CAMP2488 | 1,573,736 | chicken | 257 | ST-257 | [6] |
| 23 | CAMP3311 | 1,431,051 | duck | 3311 |  | [6] |
| 24 | CAMP828 | 1,570,976 | chicken | 828 | ST-828 | [6] |
| 25 | CAMP3136 | 1,657,253 | human |  |  | [6] |
| 26 | NC 009839 | 1,628,115 |  | 267 | ST-283 | [6] |
| 27 | NC 008787 | 1,616,554 |  | 604 | ST-42 | [6] |
| 28 | NC 003912 | 1,777,831 |  | 354 | ST-354 | [6] |
| 29 | NC_002163 | 1,641,481 |  | 43 | ST-21 | [6] |
| 30 | CAMP1044 | 1,613,621 |  |  |  | [6] |
| 32 | CampsClin11 | 1,650,105 | human | 11 | ST-45 | [7] |
| 34 | CampsClin262 | 1,643,032 | human | 262 | ST-21 | [7] |
| 36 | CampsClin266 | 1,695,272 | human | 266 | ST-21 | [7] |
| 37 | CampsClin883 | 1,667,560 | human | 883 | ST-21 | [7] |
| 39 | chick2219 | 1,616,482 | chicken | 2219 | ST-45 | [7] |
| 40 | chicka21 | 1,726,327 | chicken | 21 | ST-21 | [7] |
| 42 | cow42 | 1,672,737 | cattle | 42 | ST-42 | [7] |
| 43 | chick2253 | 2,069,686 | chicken |  |  | [7] |
| 44 | chick1717 | 1,696,727 | chicken | 1009 |  | [7] |
| 45 | chick594 | 1,609,163 | chicken | 583 | ST-45 | [7] |
| **Table S1.** continued | |  |  |  |  |  |
| **Genome ID** | **Name** | **Assembled genome size (bp)** | **Source** | **ST** | **Clonal complex** | **Reference** |
| 48 | cow206 | 1,671,619 | cattle | 206 | ST-206 | [7] |
| 49 | cow38 | 1,663,148 | cattle | 38 | ST-48 | [7] |
| 52 | cow334 | 1,616,567 | cattle | 334 | ST-45 | [7] |
| 54 | chick267 | 1,591,217 | chicken | 267 | ST-283 | [7] |
| 55 | CampsClin230 | 1,625,711 | human | 230 | ST-45 | [7] |
| 56 | cowa45 | 1,607,778 | cattle | 45 | ST-45 | [7] |
| 57 | chick2213 | 1,620,325 | chicken | 334 | ST-45 | [7] |
| 59 | cow518 | 1,705,325 | cattle | 21 | ST-21 | [7] |
| 60 | CampsClin53 | 1,658,292 | human | 53 | ST-21 | [7] |
| 62 | cowa21 | 1,658,436 | cattle | 21 | ST-21 | [7] |
| 63 | chickc21 | 1,679,349 | chicken | 21 | ST-21 | [7] |
| 64 | chick25 | 1,698,035 | chicken | 814 | ST-661 | [7] |
| 65 | chick104 | 1,761,202 | chicken | 104 | ST-21 | [7] |
| 66 | chick353 | 1,776,210 | chicken | 353 | ST-353 | [7] |
| 67 | chickb354 | 1,688,706 | chicken | 354 | ST-354 | [7] |
| 68 | chick573 | 1,838,022 | chicken | 573 | ST-573 | [7] |
| 69 | chick2568 | 1,821,236 | chicken | 2568 | ST-661 | [7] |
| 70 | chickc45 | 1,595,762 | chicken | 45 | ST-45 | [7] |
| 71 | chick19 | 1,689,713 | chicken | 50 | ST-21 | [7] |
| 72 | chick50 | 1,692,341 | chicken | 50 | ST-21 | [7] |
| 73 | chick53 | 1,651,079 | chicken | 53 | ST-21 | [7] |
| 74 | chick262 | 1,606,379 | chicken | 262 | ST-21 | [7] |
| 75 | chick266 | 1,693,845 | chicken | 266 | ST-21 | [7] |
| 77 | chick1086 | 1,692,435 | chicken | 50 | ST-21 | [7] |
| 78 | chick1360 | 1,693,941 | chicken | 50 | ST-21 | [7] |
| 79 | chick11 | 1,645,238 | chicken | 11 | ST-45 | [7] |
| 80 | chick137 | 1,734,017 | chicken | 2030 | ST-257 | [7] |
| 81 | chick1003 | 1,617,200 | chicken | 1003 | ST-45 | [7] |
| 82 | chick2048 | 1,631,119 | chicken | 45 | ST-45 | [7] |
| 83 | chick2197 | 1,667,030 | chicken | 354 | ST-354 | [7] |
| 84 | chick2223 | 1,605,483 | chicken | 45 | ST-45 | [7] |
| 85 | cow3583 | 1,654,563 | cattle | 3583 | ST-42 | [7] |
| 86 | cow618 | 1,659,558 | cattle | 61 | ST-61 | [7] |
| 87 | cow273 | 1,715,399 | cattle | 273 | ST-206 | [7] |
| 88 | cow270 | 1,716,038 | cattle | 270 | ST-403 | [7] |
| 89 | cowb21 | 1,659,711 | cattle | 21 | ST-21 | [7] |
| 90 | cowb45 | 1,603,131 | cattle | 45 | ST-45 | [7] |
| 91 | cowc45 | 1,602,224 | cattle | 45 | ST-45 | [7] |
| 92 | cowd45 | 1,607,691 | cattle | 45 | ST-45 | [7] |
| **Table S1.** continued | |  |  |  |  |  |
| **Genome ID** | **Name** | **Assembled genome size (bp)** | **Source** | **ST** | **Clonal complex** | **Reference** |
| 94 | cow104 | 1,762,939 | cattle | 104 | ST-21 | [7] |
| 96 | cow3189 | 1,894,874 | cattle |  |  | [7] |
| 97 | cow3201 | 1,629,692 | cattle | 19 | ST-21 | [7] |
| 98 | cow3202 | 1,700,098 | cattle | 827 | ST-828 | [7] |
| 99 | cow3205 | 1,720,953 | cattle | 206 | ST-206 | [7] |
| 100 | cow137 | 1,626,334 | cattle | 137 | ST-45 | [7] |
| 102 | cow583 | 1,607,515 | cattle | 583 | ST-45 | [7] |
| 103 | cow3207 | 1,641,790 | cattle | 334 | ST-45 | [7] |
| 104 | cow3214 | 1,654,338 | cattle | 45 | ST-45 | [7] |
| 105 | chick354 | 1,697,013 | chicken | 257 | ST-257 | [7] |
| 106 | chick51 | 1,714,044 | chicken | 51 | ST-443 | [7] |
| 107 | chick1079 | 1,838,061 | chicken | 1079 | ST-573 | [7] |
| 108 | chick574 | 1,743,461 | chicken | 574 | ST-574 | [7] |
| 109 | chick814 | 1,759,789 | chicken | 814 | ST-661 | [7] |
| 110 | chickb21 | 1,656,261 | chicken | 21 | ST-21 | [7] |
| 111 | chickb45 | 1,649,834 | chicken | 45 | ST-45 | [7] |
| 112 | chickd45 | 1,618,948 | chicken | 45 | ST-45 | [7] |
| 113 | chick883 | 1,665,144 | chicken | 883 | ST-21 | [7] |
| 114 | chick230 | 1,633,592 | chicken | 230 | ST-45 | [7] |
| 116 | CampsClin21 | 1,656,471 | human |  |  | [7] |
| 117 | OxClina21 | 1,697,696 | human | 21 | ST-21 | [7] |
| 119 | OxClina45 | 1,621,668 | human | 45 | ST-45 | [7] |
| 122 | starling177 | 1,582,720 | wild bird | 177 | ST-177 | [7] |
| 124 | starling45 | 1,603,669 | wild bird | 45 | ST-45 | [7] |
| 125 | starling1020 | 1,578,916 | wild bird | 1020 | ST-682 | [7] |
| 126 | goose1033 | 1,663,834 | wild bird | 1033 | ST-1034 | [7] |
| 127 | goose702 | 1,538,093 | wild bird |  |  | [7] |
| 128 | goose137 | 1,600,143 | wild bird | 137 | ST-45 | [7] |
| 129 | goose696 | 1,561,449 | wild bird | 696 | ST-1332 | [7] |
| 130 | duck702 | 1,669,953 | duck | 702 | ST-702 | [7] |
| 131 | duck45 | 1,616,162 | duck | 45 | ST-45 | [7] |
| 132 | Cc111-3 | 1,784,160 | pig | 1467 | ST-828 | [7] |
| 133 | Cc90-3 | 1,763,407 | pig | 3862 |  | [7] |
| 134 | CcZ163 | 1,676,266 | chicken | 3336 | ST-828 | [7] |
| 135 | Cc2548 | 1,855,950 | chicken | 1167 |  | [7] |
| 136 | Cc2553 | 1,822,898 | chicken | 825 | ST-828 | [7] |
| 137 | Cc2680 | 1,817,347 | chicken | 3872 | ST-828 | [7] |
| 138 | Cc2685 | 1,761,587 | chicken | 1082 | ST-828 | [7] |
| 139 | Cc2688 | 1,797,366 | chicken | 1017 | ST-828 | [7] |
| **Table S1.** continued | |  |  |  |  |  |
| **Genome ID** | **Name** | **Assembled genome size (bp)** | **Source** | **ST** | **Clonal complex** | **Reference** |
| 140 | Cc2692 | 1,764,946 | chicken | 860 | ST-828 | [7] |
| 141 | Cc2698 | 1,808,789 | chicken | 829 | ST-828 | [7] |
| 142 | Cc84-2 | 1,700,441 | pig | 1113 | ST-828 | [7] |
| 143 | Cc80352 | 1,948,336 | chicken | 1017 | ST-828 | [7] |
| 144 | Cc86119 | 1,737,119 | chicken | 825 | ST-828 | [7] |
| 145 | Cc1091 | 1,654,669 | cattle | 1068 | ST-828 | [7] |
| 146 | Cc1098 | 1,765,589 | cattle | 1104 | ST-828 | [7] |
| 147 | Cc1148 | 1,702,760 | cattle | 1068 | ST-828 | [7] |
| 148 | Cc1417 | 1,773,147 | cattle | 3221 | ST-828 | [7] |
| 149 | Cc132-6 | 1,773,147 | pig | 3221 | ST-828 | [7] |
| 150 | Cc1891 | 1,654,803 | cattle | 1068 | ST-828 | [7] |
| 151 | Cc1909 | 1,705,267 | cattle | 1104 | ST-828 | [7] |
| 152 | Cc59-2 | 1,764,317 | pig | 890 | ST-828 | [7] |
| 153 | Cc1948 | 1,703,540 | cattle | 1104 | ST-828 | [7] |
| 154 | Cc1957 | 1,826,730 | cattle | 2698 | ST-828 | [7] |
| 155 | Cc1961 | 1,770,268 | cattle | 1104 | ST-828 | [7] |
| 156 | Cc202/04 | 1,674,643 | human | 1585 | ST-828 | [7] |
| 157 | Cc67-8 | 1,856,922 | pig | 1061 | ST-828 | [7] |
| 158 | Cc317/04 | 1,751,938 | human | 5160 | ST-1150 | [7] |
| 159 | CcLMG9854 | 1,716,566 | human | 1068 | ST-828 | [7] |
| 160 | CcLMG23336 | 1,664,441 | human | 3868 | ST-828 | [7] |
| 161 | CcLMG23341 | 1,616,262 | human | 855 | ST-828 | [7] |
| 162 | CcLMG23342 | 1,623,541 | human | 855 | ST-828 | [7] |
| 163 | CcLMG23344 | 1,798,140 | human | 1586 | ST-828 | [7] |
| 164 | Cc151-9 | 1,680,175 | pig | 1102 |  | [7] |
| 165 | CcLMG9853 | 1,667,176 | human | 3869 | ST-828 | [7] |
| 166 | CcLMG9860 | 1,849,541 | human | 900 |  | [7] |
| 167 | CcH6 | 1,712,639 | human | 3020 | ST-828 | [7] |
| 168 | CcH8 | 1,804,264 | human | 901 | ST-828 | [7] |
| 169 | CCH9 | 1,673,028 | human | 825 | ST-828 | [7] |
| 170 | CcH56 | 1,737,522 | human | 1096 | ST-828 | [7] |
| 171 | CcZ156 | 1,716,763 | chicken | 854 | ST-828 | [7] |
| 172 | Cj129-258 | 1,643,841 | cattle | 459 | ST-42 | [7] |
| 173 | Cj51494 | 1,799,590 | chicken | 4834 | ST-353 | [7] |
| 174 | CjLMG23216 | 1,708,741 | chicken | 4835 |  | [7] |
| 175 | CjLMG23218 | 1,677,541 | chicken | 48 | ST-48 | [7] |
| 176 | CjLMG23223 | 1,701,738 | chicken | 791 |  | [7] |
| 177 | CjLMG23263 | 1,739,638 | chicken | 3504 | ST-446 | [7] |
| 178 | Cj60004 | 1,674,182 | chicken | 4836 |  | [7] |
| **Table S1.** continued | |  |  |  |  |  |
| **Genome ID** | **Name** | **Assembled genome size (bp)** | **Source** | **ST** | **Clonal complex** | **Reference** |
| 179 | CjLMG23264 | 1,719,111 | human | 46 | ST-206 | [7] |
| 180 | CjLMG23269 | 1,735,582 | chicken | 4837 | ST-353 | [7] |
| 181 | Cj55037 | 1,598,300 | chicken | 45 | ST-45 | [7] |
| 182 | CjLMG9879 | 1,650,086 | human | 47 | ST-21 | [7] |
| 183 | Cj86605 | 1,637,051 | chicken | 4840 | ST-48 | [7] |
| 184 | CjLMG23357 | 1,694,896 |  | 4883 | ST-1275 | [7] |
| 185 | CjATCC33560T | 1,714,676 | cattle | 403 | ST-403 | [7] |
| 186 | CjLMG9081 | 1,593,688 | human | 52 | ST-52 | [7] |
| 187 | Cj53161 | 1,730,741 | chicken | 4838 | ST-353 | [7] |
| 188 | CjLMG9217 | 1,663,474 | human | 443 | ST-443 | [7] |
| 189 | Cj2008-1025 | 1,656,768 | human | 50 | ST-21 | [7] |
| 190 | Cj2008-894 | 1,627,102 | human | 1962 |  | [7] |
| 191 | Cj2008-872 | 1,605,881 | human | 61 | ST-61 | [7] |
| 192 | Cj2008-988 | 1,818,384 | human | 572 | ST-206 | [7] |
| 193 | Cj1997-1 | 1,603,628 | human | 658 | ST-658 | [7] |
| 194 | Cj2008-979 | 1,798,587 | human | 2274 |  | [7] |
| 195 | Cj2008-831 | 1,606,170 | human | 50 | ST-21 | [7] |
| 196 | Cj1997-4 | 1,669,690 | human | 475 | ST-48 | [7] |
| 197 | Cj1997-7 | 1,633,444 | human | 61 | ST-61 | [7] |
| 198 | Cj1997-10 | 1,791,227 | human | 4839 |  | [7] |
| 199 | Cj1997-11 | 1,598,427 | human | 22 | ST-22 | [7] |
| 200 | Cj1997-14 | 1,767,166 | human | 5159 | ST-353 | [7] |
| 201 | Cj51037 | 1,747,053 | chicken | 939 | ST-353 | [7] |
| 202 | Cj110-21 | 1,615,152 | cattle | 982 | ST-21 | [7] |
| 203 | Cj87330 | 1,618,859 | chicken | 50 | ST-21 | [7] |
| 204 | Cj87459 | 1,772,922 | chicken | 452 | ST-353 | [7] |
| 205 | Cj140-16 | 1,677,874 | cattle | 5161 | ST-61 | [7] |
| 206 | Cj1213 | 1,673,339 | cattle | 132 | ST-508 | [7] |
| 207 | CjATCC43432 | 1,703,446 | human | 122 | ST-206 | [7] |
| 208 | Cj1798 | 1,604,776 | cattle | 61 | ST-61 | [7] |
| 209 | Cj1854 | 1,619,643 | cattle | 922 |  | [7] |
| 210 | Cj1893 | 1,700,567 | cattle | 38 | ST-48 | [7] |
| 211 | Cj1928 | 1,744,799 | cattle | 806 | ST-21 | [7] |
| 212 | CjLMG9872 | 1,619,961 | human | 677 | ST-677 | [7] |
| 213 | Cj23210 | 1,762,534 | chicken | 380 |  | [7] |
| 214 | CjLMG23211 | 1,669,921 | chicken | 220 | ST-179 | [7] |
